# The impact of acute adenolymphangitis in podoconiosis on caregivers: A case study in Wayu Tuka woreda, Oromia, Western Ethiopia. ‘*If she was healthy*, *I would be free*.’

**DOI:** 10.1371/journal.pntd.0007487

**Published:** 2019-07-08

**Authors:** Clare Phillips, Abdi Samuel, Gemechu Tiruneh, Kebede Deribe, Gail Davey

**Affiliations:** 1 Department of Global Heath and Infection, Brighton and Sussex Medical School, Falmer, Sussex, United Kingdom; 2 College of Medical and Health Sciences, Wollega University, Nekemte, Ethiopia; 3 School of Public Health, College of Health Sciences, Addis Ababa University, Addis Ababa, Ethiopia; RTI International, UNITED STATES

## Abstract

**Background:**

Podoconiosis, also known as mossy foot or endemic non-filarial elephantiasis, is a preventable form of lower-leg lymphoedema caused by prolonged (typically barefoot) exposure to soil derived from volcanic rocks. Acute adenolymphangitis (also called ‘acute attack’) is a serious complication of podoconiosis resulting in significant symptoms and worsening disability. Despite the well-known morbidity associated with podoconiosis, to date there have been no studies looking at the impact, or burden, of podoconiosis on caregivers. This study explored the experiences and impact of acute attacks on the caregivers of those with podoconiosis in one endemic district of Ethiopia.

**Methods/Principal findings:**

This qualitative study was based in Wayu Tuka *woreda* (district), Oromia, Western Ethiopia. 27 semi-structured interviews of those with podoconiosis and their caregivers were conducted in June 2018. Here we report the findings from the caregiver’s interviews. Data were analysed using NVivo 12. Directed content analysis, a qualitative approach related to thematic analysis, was used to analyse the results. This study highlights a previously unreported impact of acute attacks on the caregivers of those affected by podoconiosis. The findings demonstrate the significant social and financial pressures placed on podoconiosis-affected families which are exacerbated during acute attacks. This study also highlighted the emotional burden experienced by caregivers, the range of care activities placed on them and the limited support available.

**Conclusions:**

This study found a significant impact on the caregivers of those with podoconiosis, especially during acute attacks, in Wayu Tuka *woreda*. It also highlighted the limited support available to caregivers. Further research is needed to understand whether this impact applies to podoconiosis caregivers across Ethiopia, and beyond, and to establish if there are wider implications of this important consequence of podoconiosis, for example on the economy and caregivers’ mental and physical health.

## Author summary

Podoconiosis is a foot condition, common in the highlands of Ethiopia, caused by exposure to volcanic soil. It can be prevented by wearing shoes and adhering to foot hygiene practices. Podoconiosis causes swelling of the lower legs and is a disabling and stigmatised condition. It is made worse by ‘acute attacks’, during which the leg becomes painful, swollen and red. Often the person affected cannot work and is bedbound during these episodes. This study identified a previously unreported burden on the caregivers of those with podoconiosis during acute attacks in one endemic district of rural Ethiopia. Specifically, we identified a significant social and financial pressure placed on podoconiosis-affected families in meeting healthcare costs, covering daily expenses such as children’s education costs and progressing within their communities. This study also highlighted the emotional burden experienced by caregivers, the range of care activities placed on caregivers and the limited support available. These findings warrant further research in other contexts but highlight an important wider social consequence of podoconiosis.

## Introduction

Podoconiosis, also known as mossy foot or endemic non-filarial elephantiasis, is a preventable form of lower-leg lymphoedema caused by exposure to soil derived from volcanic rock [1, 2]. It is most prevalent where volcanic soil is found together with altitudes >1000m above sea level and rainfall >1000mm/year [3]. Podoconiosis mainly affects poor, bare footed agricultural communities, who cannot afford protective footwear and who do not have access to clean water for washing [4, 5]. Although podoconiosis is only found in individuals exposed to these volcanic soils, familial clustering is common, and a genetic link has been identified [6]. Disease progression can be halted by adhering to foot hygiene practices [7, 8].

Like many Neglected Tropical Diseases (NTDs), podoconiosis rarely causes mortality, but instead causes disabling changes to the lower legs, affecting mobility, quality of life and economic productivity [8, 9]. Podoconiosis onset is most commonly reported between 16 and 45 years of age, affecting the most economically active groups [10, 11]. Additionally, podoconiosis is highly stigmatised, with evidence of those affected being excluded, insulted or shunned by their communities [1, 2, 12, 13].

Podoconiosis has been identified in 32 countries, making it a health problem of global concern [14]. In 2011, it was recognised as one of the NTDs by the World Health Organisation (WHO), under the category of ‘other tropical conditions’ [15]. Ethiopia is thought to have the highest number of podoconiosis cases of any country; here it is highly endemic with recent mapping suggesting 1,537,963 adults are living with podoconiosis and a further 34.9 million are at risk of the disease [3, 16]. Podoconiosis is endemic in 345 of Ethiopia’s 839 *woreda*s [3, 4].

Acute adenolymphangitis, or acute dermatolymphangioadenitis (ADLA), known commonly as ‘acute attacks’, is the term used to describe recurrent inflammatory episodes in lymphoedema [17]. ADLA is most likely triggered by skin lesions acting as entry points for bacteria, fungi or viruses, although often no clear cause is identified [18,19]. Episodes are typically characterized by hot, painful and reddened swelling of the leg, chills, malaise, anorexia and sometimes lower leg skin peeling, and are one of the most serious complications of podoconiosis; resulting in worsening disability [8, 20].

ADLA incidence varies across the studies conducted to date in Ethiopia, ranging from 5.5 to 23.3 episodes per person per year, with each episode lasting on average 6.42 days [21, 22]. As ADLA leaves individuals’ bed-bound, it is estimated that anywhere between 24 and 149.5 working days are lost per year [8, 21, 22].

While there is no panacea for ADLA, previous research in Ethiopia observed that those participants who never walked barefoot and who engaged in daily foot washing had lower odds of developing ADLA [21]. The GoLBeT study, a randomised controlled trial conducted in Northern Ethiopia, evaluated the impact of a simple lymphoedema care package (including foot washing and shoe wearing) on the incidence of ADLA in those with podoconiosis [8]. It found that incidence of ADLA was lower in the intervention group at 19.3 episodes per person year compared to 23.9 episodes per person year in the control group [8]. Additionally, the duration of attacks was found to be shorter in the intervention group [8].

State-provided or private care is not available or is unaffordable to the majority living with disabilities in LMICs, and most care is therefore provided by unpaid family or friends; often referred to ‘family caregivers’ or ‘informal caregivers’ [23]. In 2015 the WHO defined a caregiver *as ‘a person who provides care and support to someone else; such support may include*: *helping with self-care*, *household tasks*, *mobility*, *social participation and meaningful activities; offering information*, *advice and emotional support*, *as well as engaging in advocacy*, *providing support for decision making and peer support*, *and helping with advance care planning; offering respite services; and engaging in activities to foster intrinsic capacity* [24].’

Whilst caregiving may be rewarding, can strengthen family ties or be part of honouring the person needing care; many caregivers struggle with the burden of caregiving [25]. ‘Caregiver burden’ is broadly summarised as *‘the extent to which caregivers perceive that caregiving has had an adverse effect on their emotional, social, financial, physical, and spiritual functioning [25].’* We have used this definition of ‘caregiver burden’ throughout this study.

NTDs, such as podoconiosis, are widely known to cause morbidity and disability more frequently than mortality; typically, YLD (years lived with disability) greatly outweigh YLL (years of life lost) in DALY (disability-adjusted life years) calculations for NTDs [26, 27]. Despite evidence of high disability amongst those affected by NTDs, and the disproportionate impact of NTDs on LMICs, there is a surprising dearth of literature on the burden of NTDs on caregivers in these regions. A review of NTDs and mental health in 2012 found no papers on the impact of NTDs on caregivers [28]. A 2014 review of the literature on the burden of caregiving for those affected by disability or disease in LMICs, highlighted a lack of published data, with most research conducted focusing on HIV/AIDS and mental ill-health [23].

An economic evaluation of podoconiosis in 2006 reported that caregivers lost an average of 9 work days per quarter through supporting relatives with podoconiosis to clinics or caring for them at home, equivalent to an opportunity cost of 4224 birr (approximately $350) [11]. Beyond this, no study to date has explored the burden, or impact, of podoconiosis and ADLA on caregivers.

## Methods

This study explored the experiences and impact of acute attacks on the caregivers of people with podoconiosis in Wayu Tuka *woreda*, an endemic district of western Ethiopia.

### Study design

We used qualitative research methodology to allow participants to describe their experiences in their own words in order to draw out unanticipated or culturally relevant answers [29, 30]. Content analysis, related to thematic analysis, was used. A cross-sectional exploratory study, in one geographical area, was used to allow for holistic, in-depth investigation in a small, real-life context.

### Ethical approval

Ethical approval was obtained from the Research Governance and Ethics Committee (RGEC) at Brighton and Sussex Medical School (BSMS) in January 2018 and from Wollega University Institutional Review Board (IRB) in March 2018 prior to initiation of the study.

### Study site

The study was conducted at Konchi clinic, in Wayu Tuka *woreda* which lies in East Wollega zone in the state of Oromia, Western Ethiopia. The clinic sits just off the main tarmac road, approximately 16km from Nekemte, and 7km from Gute, the main town in Wayu Tuka. A study in 2016 reported a high incidence of ADLA in Wayu Tuka *woreda*, with participants on average experiencing 23.3 episodes annually, making this an ideal setting in which to conduct this study [21]. Konchi clinic is run by an order of Indian Catholic nuns who provide, amongst other services, free leg washing to those affected by podoconiosis.

### Participant recruitment and sampling

Caregivers were identified by those affected by podoconiosis who attended the clinic for leg washing, using snowball sampling [29]. Snowball sampling relies on participants to invite or identify others who may be willing to participate in the study [31]. No relationship existed between the principal researcher (CP) and clinic staff, those attending leg-washing services or caregivers prior to recruitment. Those affected by podoconiosis were selected using purposive sampling, specifically, maximum variation sampling was used to recruit participants with podoconiosis from across the disease spectrum, in order to understand the impact and experiences of those with all stages of the disease [32]. Participants were provided with a Participant Information Sheet (PIS), in Afaan Oromo, informing them of the aims and purpose of the study. This was read to participants who were unable to read. Participants were recruited (face-to-face) if they met the inclusion criteria (see Table 1). Informed, written consent was obtained from all adult participants. Informed, written assent was obtained from participants over 12 years of age and the informed written consent of their parent or guardian was also obtained. A thumb print was obtained from those who could not write. A total of 13 caregivers were included in the study. No one who was approached declined recruitment.

**Table 1:** Inclusion/exclusion criteria.

Inclusion criteria—Caregiver	Inclusion criteria—Patient
All genders	All genders
Resident of Wayu Tuka *woreda*	Resident of Wayu Tuka *woreda*
Participant feels well enough to take part in the interview	Participant feels well enough to take part in the interview
Those identified as a caregiver by the person with podoconiosis	All people diagnosed with podoconiosis by a health worker (irrespective of disease stage) and who report experience of at least one acute attack at any time
Participant has capacity and is willing to give informed consent and is aged >18 years **OR** >12 years old, able and willing to give assent and parent/guardian able to provide informed consent	Participant has capacity and is willing to give informed consent and is aged >18 years
Exclusion criteria	
Those who do not have capacity or who are not willing to provide informed consent/assent	

### Data collection

Data was collected via semi-structured interviews, using a topic guide developed by the principal researcher that was translated into Afaan Oromo. A meeting was held at Wollega University, before recruitment began, with the qualitative interviewer, research assistant, study supervisor and the principal researcher to discuss the topic guides in detail, agree and adjust the translated versions of the topic guides and agree definitions and local terms for ADLA and caregivers. Locally, ADLA is referred to *‘dhukuba muddaa’* or *‘nyaataa muddaa’*. A primary caregiver was defined as ‘a friend/relative/member of the community who provides the majority of care to the participant. Care activities include assistance with personal care e.g. washing, dressing, toileting and eating, household activities e.g. food preparation, cooking, shopping, laundry, collecting water and cleaning, medical care e.g. obtaining and administering medication, accompanying to appointments, wound care, monitoring patient’s medical condition, physical assistance e.g. assisting the person to walk, moving or carrying the person, transport/mobility outside of the home, emotional support or assistance [23, 33]. The topic guide included open questions around the impact of acute attacks on daily life, support available to caregivers, understanding of symptoms, prevention and causation and access to healthcare. Face to face interviews were conducted in Aafan Oromo by an experienced, qualitative researcher working as a lecturer in the School of Public Health at Wollega University. Interviews were audio recorded on two password-protected devices to ensure they were accurately transcribed, and a high-quality recording was obtained. Interviews ranged from 15–30 minutes in duration.

Data collection took place at Konchi clinic from 5-7th June 2018. Those with podoconiosis either waited to be interviewed or returned at an appointed time on subsequent days. Caregivers were asked by their affected relative to attend the clinic for interview at a convenient or pre-arranged time. Interviews were held in an office at the clinic and were conducted until data saturation was reached. Caregivers were interviewed separately to the person they cared for. Data saturation was determined by the interviewer, following discussion of each interview’s content with the principal researcher.

### Data analysis

Interviews were first transcribed to Microsoft Word in Afaan Oromo from the audio recordings by an audio-typist at Wollega university. The Afaan Oromo transcripts were then read, sections of the audio recordings listened to for confirmation of the transcript, and verbally translated into English by the study’s research assistant (GT). The principal researcher (CP) directly transcribed the English verbal translation to Microsoft Word, aiding data immersion [31]. Finally, one third of the translated interviews were checked by an impartial lecturer in Public Health from Wollega University, who listened to the audio recording in Afaan Oromo whilst reading the English transcription. This was to establish the accuracy of the translations.

**Fig 1:**
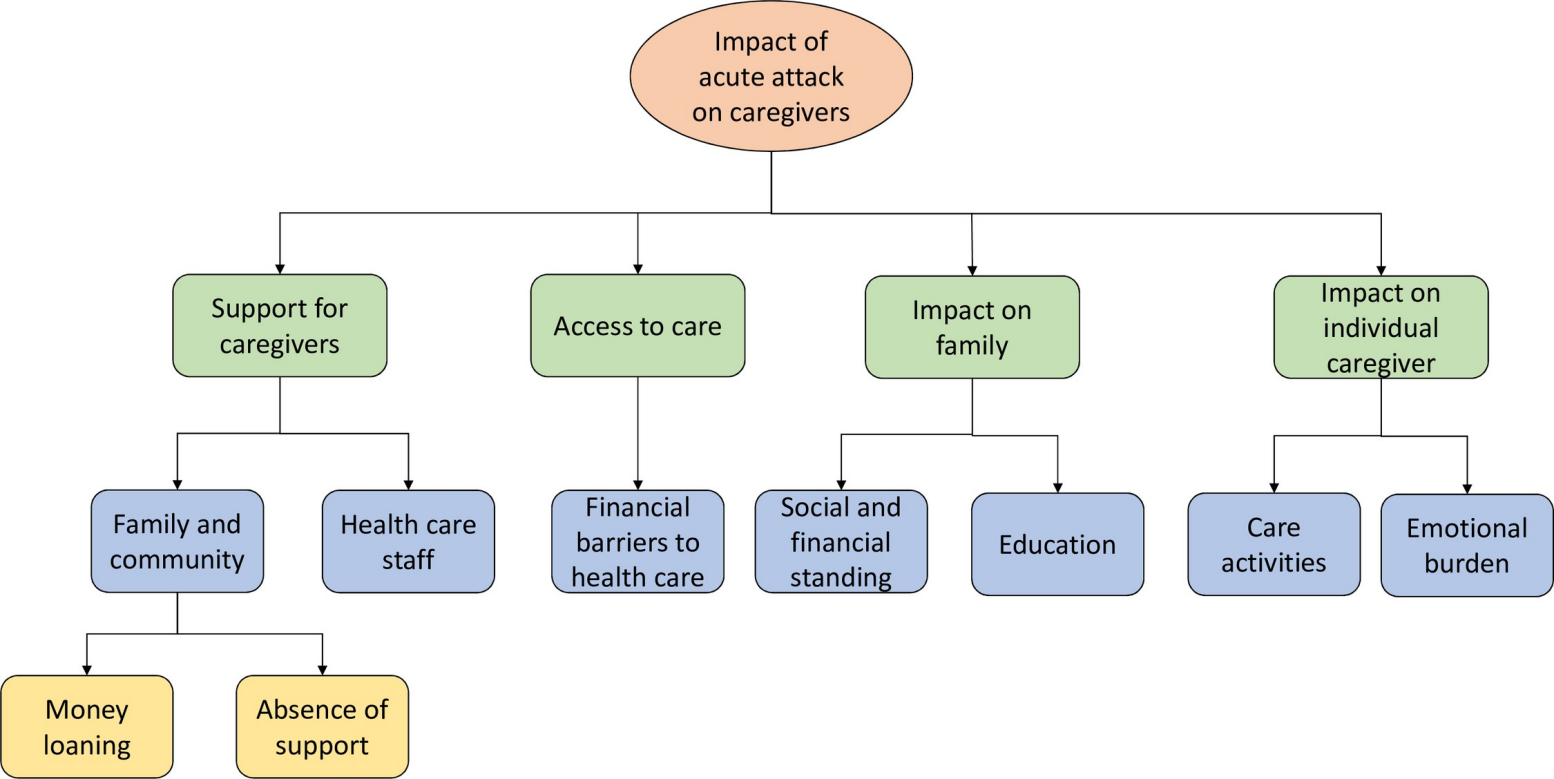
Themes and subthemes identified.

On return to the UK, interviews were uploaded to NVivo 12 which was used to aid the coding process. Directed content analysis, a qualitative approach related to thematic analysis, was used to analyse the results [31, 34]. Data were completely coded (by CP) to broad themes that were created from previous research and which were used to structure the interview topic guides e.g. access to healthcare, impact on livelihood, education and household income [31, 34]. After this, the codes were refined and revised, and themes specific to the data were identified [34], see Fig 1.

## Results

13 caregivers were interviewed for this study. Caregivers ranged from 16–55 years of age, and the average age was 31 years. 38% of caregivers were female, 92% worked full time, and 77% described themselves as either farmers or daily labourers. A minority, 23%, had no formal education. All caregivers were family members. All participants were wearing shoes.

### Impact on family

#### Education

Impact on education was evident, with participants reporting children missing school either to perform caring duties or as a consequence of insufficient funds to cover schooling and healthcare costs.

‘***If the costs needed for education are not reduced and I don’t have the amount of money I need for her medical costs*, *I have to stop educating my children*.*’* (14cg)**

‘***I have to miss school days to help him during the attack*, *to stay at home with him…*. *Actually*, *during the attack*, *I may get exhausted because of assisting him*. *Then I go to bed without studying*.*’ (*06cg)**

#### Social and financial standing

Acute attacks commonly meant those affected were unable to work whilst ill and, at times, caregivers remained at home to care for them. This had a significant impact on livelihood and the ability of the family to earn a wage.

‘***How can I go for work*, *leaving someone who is ill alone*? *Unless I just tell the children to be with her*, *then I go out to earn a wage*.*’* (10cg)**

‘***She may be able to do work when she is ok*, *but when the problem worsens she becomes bedridden*.*’* (05cg)**

The lack of family finances was linked to the inability of families to build resources or cover daily costs. Some participants reported having to sell cash crops or cattle to meet medical expenses. Others were unable to afford necessities such as clothing.

‘***So much money is needed*, *there is a time where I sold cattle in the garden*. *If our cash crop is better one year*, *we sell that one to take her there*** *(health centre)***. *If the produce is low*, *we sell our cattle to take her to the clinic seeking her relief*.*’* (14cg)**

‘***The impact that this care causes on me is in terms of working and accumulating resources and it impacts how we can improve*. *We spend our resources on treatment*, *we can’t even change our clothes once a year*, *because even the money we should use to buy things for our children is being spent on treatment*. *So*, *we are unable to even change our clothes*.*’* (03cg)**

The inability to build resources meant caregivers and families felt left behind; many commented they could not keep up with or compete with friends and neighbours. Caring for their affected relative was holding them back from being able to progress, both financially and socially.

‘***My friends have built their own homes and are living there but because of her problem I have been investing what I earn on her*, *so we can’t advance our life*. *I can’t even teach our children*. *If she didn’t exist*, *this wouldn’t happen*. *This has really impacted us greatly*.*’* (02cg)**

‘***When she is ill*, *I always think about her feet*, *we can’t keep up with our other healthy friends*. *So*, *the impact is great when she gets ill…*. *We get angry because we can’t be like our healthier friends*.*’* (10cg)**

### Impact on individual caregiver

#### Emotional burden

**Fig 2:**
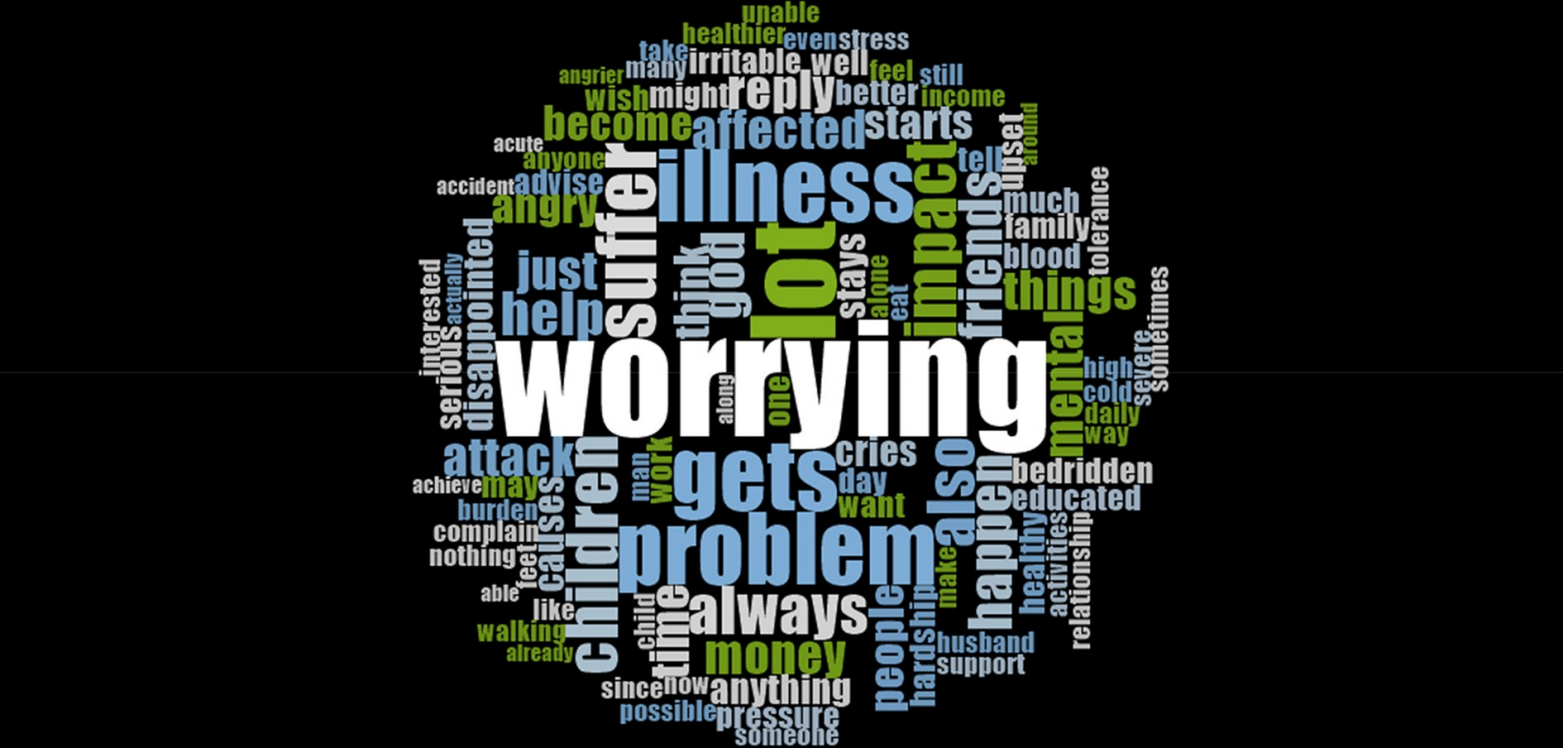
Word cloud of terms used by caregivers in relation to the theme of emotional impact.

Caregivers most commonly described the emotional impact of caring for their relative in terms of ‘worry’ (see Fig 2). Emotional distress related to finances, being able to provide for their relative and concern for the health and recovery of the affected relative.

‘***Yeah*, *mentally I am affected*. *I don’t have hope that she will be better when the attack happens to her*. *When the attack happens*, *I cry so much as well when I am unable to do anything for her*. *These things are affecting me a lot*, *me myself*.*’* (11cg)**

‘***We always worry a lot about her*, *thinking about what will happen to her*. *Will she be ok*, *or will it get worse*? … *We worry because our capital doesn’t align with the services required*.*’* (04cg)**

‘***When she is ill, I am also affected. If there is no help, it is obvious there is hardship***. ***I am suffering with her***.’ ***(10cg)***

‘***I worry a lot when these attacks start***. ***Man is for man, one is helpless if they are alone***.’ ***(03cg)***

#### Care activities

Caregivers were providing a wide range of care needs for their affected relative. Care not only consisted of physical care tasks, such as leg washing and feeding, but also involved supporting and advocating for the patient, delegating care tasks to others, decision-making on behalf of the patient and the family, supporting treatment adherence and providing financial support.

‘***When she is ill she feels cold and at that time I add warm and thick clothes over her*. *Whenever it’s possible for us*, *we give her not only things ordered by the clinic*, *we heat bakanisa leaves*** *(local leaf used to apply to cuts)* ***and apply to her body where she feels the pain*, *for the headache and where there is itching we use bizana leaves to give relief*. *We even do this if the attack is in the evening or the middle of the night*. *If its day or the morning*, *we immediately take her to the clinic* … *When her bladder is full*, *we may help her to go out or we provide her with a bowl*, *if she is incapable*.*’* (14cg)**

‘***There is soap and I apply that soap to a piece of cloth I made by hand specifically that I use to wash his leg*, *and I wash him with that*.. *When he gets up in the morning*, *he doesn’t wash*. *When he stays out in the field and comes home I wash his leg for him as well as when he is about to sleep at night’* (6cg)**

### Support for caregivers

#### Family and community

**Fig 3:**
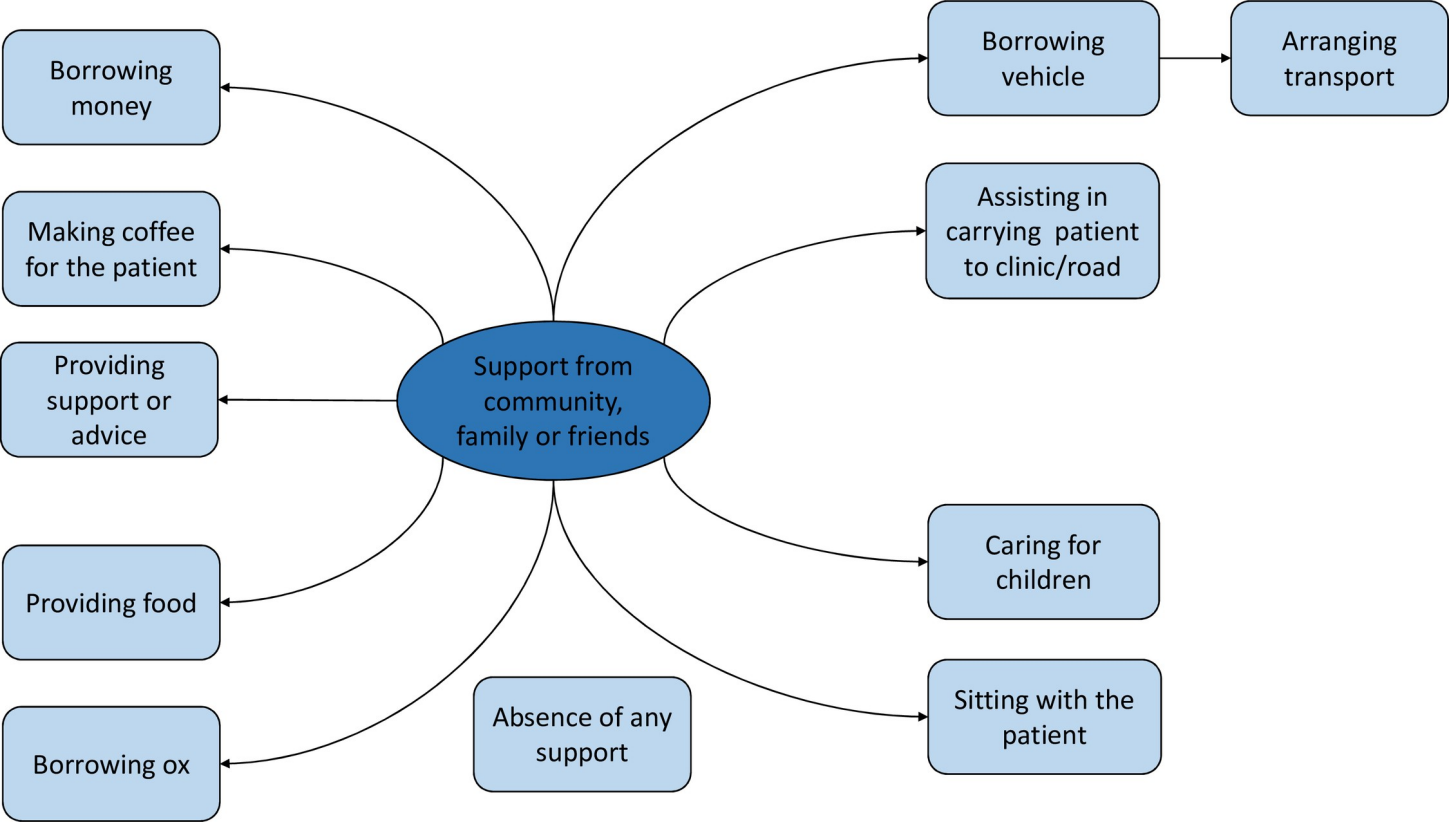
Support for caregivers from family and community.

Support from family and the community during attacks came in different forms (see Fig 3). Participants reported receiving food or meals, borrowing oxen or vehicles and some reported sending children to live with other members of the community or family.

‘***When this problem starts I beg my neighbours to make coffee for her*.*’ (*02cg)**

Additionally, caregivers reported seeking advice or support from friends, family and neighbours during the acute attacks:

***‘Her friends advise her and us to be open to them***, ***not to hide things from them***, ***to tell them every trouble***. ***On my part***, ***I try my best***, ***if I have a problem I tell my friends*** …***’* (03cg)**

‘***Yes*, *if there are problems*, *I discuss with his brothers and then they may send him something*.*’* (06cg)**

Commonly, neighbours were called on to assist in carrying the patient to clinic for treatment:

‘***If she can walk she can go*, *if not we seek for assistance from others to carry her to the health facility*.*’* (10cg)**

***‘The support I get from my friends is probably when she is severely ill and unable to walk***. ***I seek assistance from the village to help me carry her***. ***Even sometimes when she’s ill we carry on a chair and bring her to the clinic***.***’*(14cg)**

#### Money loaning

A consistent phenomenon within the theme of *family and community* was the borrowing of money from neighbours or family to access healthcare. While community money loaning schemes operate in this part of Ethiopia (e.g. *Idir*, *Equub* and *Senbat*) they were not mentioned by participants in this study. Money loaning, reported here, seemed to be cooperative and informal and was reported by nearly all participants. It appeared to be a common support mechanism within the community.

‘***At the time when there are no cents*, *we borrow from the village and take her to the clinic*.*’* (08cg)**

‘***If she is ill*, *even if I don’t have*** *(money)***, *I borrow from someone else and take her to the clinic’* (12cg)**

#### Absence of support

Furthermore, within this theme, many caregivers reported an absence of support, of being the sole caregiver and not having anyone to call on for help:

‘***We have no one to seek advice from*. *We have no one to help us*.*’* (04cg)**

‘***No one helps us*, *who can help us*? *I don’t have any other relative or any other person to help me*.*’* (05cg)**

‘***Apart from my own hand*, *no one is there to help her*.*’* (11cg)**

#### Healthcare staff

Although not directly reported as offering support by caregivers, all respondents were regularly attending the clinic for advice and treatment during acute attacks. Many reported attending on numerous occasions to seek care for their relative. The clinic was typically the first, and main, port of call during an acute attack and provided caregivers with advice and care instructions for their relatives.

‘***The first thing is*, *I take her to the clinic*. *This woman is very ill*, *and I have to support my wife*. *This problem happens every 2 weeks or every month*, *I just take her to the clinic at that time because the pain is severe*, *and she can’t tolerate this when it starts*.*’* (02cg)**

#### Access to care

Participants were asked about their experiences of accessing care, where they went for care and any barriers to this. Care was regularly accessed at the local clinic (where interviews were conducted) with respondents occasionally seeking care at other health facilities in nearby towns. Caregivers reported frequent use of services at the clinic during acute attacks and affected relatives were often attending the clinic to access the (free) leg washing services.

#### Financial barriers to healthcare

Overwhelmingly, the ability to pay was the only barrier reported in accessing healthcare. It was directly related to delayed presentation to healthcare and needing to borrowing money from others. It was also a significant cause of concern and worry for caregivers, as mentioned previously.

‘***There is no money in my hand to take her to a health facility from time to time*. *If you have no money in your hand at that time*, *there is no other mechanism like credit and I have no hope that she can be treated for free today and I can pay them back tomorrow*.*’* (02cg)**

‘***What can I do*? *She is bedridden most of the time*, *especially when the attack happens until I take her for an injection*. *In fact*, *to take her for an injection*, *I am a daily labourer*, *I don’t have enough money to take her for an injection when it happens*.*’* (05cg)**

‘***When she gets ill and if have no money at that moment*, *she just stays bedridden’* (08cg)**

The frustration of this was also expressed:

‘***I spent my money seeking health for her*, *but she is not cured*.*’* (14cg)**

## Discussion

Caregiver burden, defined in this study as *‘the extent to which caregivers perceive that caregiving has had an adverse effect on their emotional, social, financial, physical, and spiritual functioning [25]’,* was evident in the caregivers of those affected by podoconiosis and ADLA in Wayu Tuka *woreda*.

Consistently, caregivers reported the significant financial impact of acute attacks on covering healthcare costs and the pressure these recurrent costs placed on family finances for other necessities such as clothes, food and children’s education. The ability to earn a wage was not only restricted in those directly affected by podoconiosis and ADLA but also in caregivers, who remained at home to care for them. Furthermore, resources that caregivers were able to accumulate were being periodically drained to cover healthcare costs, preventing caregivers, and podoconiosis-affected families, from being able to progress, or keep up with, others in their community. The emotional worry caregivers reported related directly to the financial pressure they were placed under, as well as concern for their affected relative. These findings are supported by those of Perera et al (2007) in participants with lymphatic filariasis (LF) in Sri Lanka [35]. Participants reported the devastating impact of LF on household income–this comprised of loss of earnings through their inability to work, loss of earnings of family members who stayed at home to care for them or carry out other household tasks and the burden of recurrent medication costs [35]. Children, as in our study, were held back from school to help with work. The notion of being unable to progress was also noted; ‘*for some participants*, *the presence of a member with LF had been a hindrance to family progress*, *rather than a cause of poverty*, *holding the family finances back when they could have achieved an improved standard of living*’ [35].

It was apparent from our study that, despite clear recognition of when healthcare was required and trust in the local clinic, participants simply could not afford to pay the required costs for medicines needed during an episode of ADLA. Potentially compounding this was the very high incidence of attacks reported by caregivers. Occurring as frequently as every 2–3 days, with an average of 6 attacks per month (see supporting information), the incidence in this cohort was 3-4-fold that reported in other studies in the region [21, 22]. It is unclear why incidence was so high in this cohort and further evaluation is required. This study has confirmed the debilitating healthcare costs families face as a consequence of podoconiosis [11, 36]. Financial risk protection for those affected by podoconiosis and health financing of podoconiosis treatment needs to be reviewed, as the current NTD Master Plan fails to comprehensively account for this [4].

Burden on caregivers has been linked to increased mental illness, particularly depression, in those caring for relatives with HIV, neurological and mental health conditions [28]. The burden of depression in caregivers of those with LF has been theoretically estimated at 229,537 DALYs and the caregivers of those with cutaneous leishmaniasis are known to have significantly higher rates of depression and lower quality of life [37, 38]. A decline in mental health has also been linked to a decline in physical health amongst caregivers [28, 39]. A review of NTDs and mental health commented that ‘*There appears to be no research on the impact of NTDs on the mental health of caregivers who play a significant role in support of the sufferers of those burdened by NTD lesions and sequelae. The significance of the role of caregivers is a subject that requires study given the needs of patients who require almost constant and lifelong care [28].’*

While the impact of caregiver burden on mental and physical health was not assessed in this study, it is important that caregiver burden in podoconiosis and its consequences are measured. Caregivers in this study were in the economically active age group, the average age being 31 years. The hidden costs of caregiver burden are therefore an important consideration in future calculations of the economic, as well as the social, impact of podoconiosis.

### Support for caregivers

Support from friends, neighbours and healthcare staff was evident in those attending the leg washing clinic in Wayu Tuka, however the absence of support was a consistent theme reported by participants in this study. This contradiction, particularly in relation to the support offered by healthcare staff, most likely relates to the support patients expected and what the clinic staff were able to offer. The clinic had not received any funding for podoconiosis care for a number of years at the time of the study and all care was provided by charitable donations to the order. Caregivers have been found to report lower burdens when they receive more social support, yet with changes in traditional societies, support mechanisms are thought to be declining in LMICs [23, 39]. This would require further evaluation in this context.

One relatively novel finding was the borrowing of money from neighbours and friends to pay medical costs; money loaning was reported by nearly all participants in the study. This appeared to be outside of known money loaning schemes such as *Idir*, *Equub* and *Senbat*, which operate in this region of Ethiopia. Whilst money loaning has not been directly reported in studies on podoconiosis in Ethiopia, a study in Cameroon found podoconiosis patients spent 142 USD per year on direct healthcare costs, of which 34USD was borrowed from friends, family and community groups [36]. Although money loaning in this study was reported as the social norm, one participant reported knowing people in debt who had killed themselves. This again highlights the need for appropriate healthcare financing of healthcare costs in those affected by podoconiosis.

Whist the literature on caregivers in LMICs is minimal, what literature there is highlights a clear need for greater support for caregivers. Longitudinal burden measurements of the caregivers of those with bipolar disorder in southern Ethiopia concluded that more needed to be done from a health policy perspective to lessen the economic and family burden of caregivers [40].

While this study has shed light on an important yet neglected issue, there are a number of limitations. Firstly, a significant limitation to this study was that the main research assistant, who translated the interviews into English, was not experienced in qualitative research and therefore could not conduct the interviews. Whilst reasonable adjustment for this was made (the research assistant was present at the clinic during interviews and was involved in translation of topic guides, PIS and all other materials related to the study and so was highly familiar with the content) it is possible that some nuance of the interviews was lost as they were not translated by the interviewer.

Secondly, a clear error in the study was the absence of field notes. No notes about behaviour or emotions were documented. At times participants became tearful, and whilst this was acknowledged at the time, it was not documented and is a disappointing gap in the data.

Thirdly, whilst the study identified some novel and, potentially valuable, findings, due to the case-study design these are not generalisable to the whole of Ethiopia. Additionally, this study only recruited those linked to the free leg washing clinic and therefore does not reflect the experiences of those without access to this vital service. The study does however highlight areas of potential future research and supports findings from studies in other regions of the country.

Finally, the ethical approval for this study was only for those aged 12 years or over. Participants under the age of 12 were turned away from participating in this study which highlights that children are indeed acting as primary caregivers in Wayu Tuka. It was also reported in the interviews that children were kept from school to attend sick relatives. Further research into the role of children as caregivers would be useful to evaluate the full extent of this issue and its impact.

In conclusion, acute attacks have a debilitating impact on the caregivers of those with podoconiosis in Wayu Tuka *woreda*. In the context of podoconiosis and NTDs, this burden on caregivers appears to be a largely unrecognised and novel finding yet supports data from caregiver studies in other contexts and is perhaps unsurprising [23]. Whilst this is a case-study, in one small corner of Ethiopia, and has limited generalisability, it highlights some gaps in the current literature and in national policy which may be important.

Reducing the incidence of ADLA is a key priority in alleviating caregiver burden and lessons can be taken from the GoLBeT study [8]. Beyond this, further research is needed to further evaluate this important finding. Validation of a caregiver burden assessment scale for use across all NTDs, including podoconiosis, would be a useful tool to formally measure caregiver burden. The Family Burden Interview Schedule (FBIS) or the Burden Assessment Schedule (BAS), both developed in India to assess burden in the caregivers of those with mental illness, may be suitable for adaptation [41, 42]. Secondly, an economic evaluation of caregivers in podoconiosis, including consideration of mental and physical health consequences on economic productivity would be helpful in quantifying the wider implications of caregiver burden. In addition to this, the development of a cost-effective intervention to support caregivers and an evaluation of the role of children as caregivers in podoconiosis is needed. Finally, improved health financing to prevent podoconiosis-affected families falling into debt and alleviating caregiver burden is warranted. A possible mechanism for is to include expanding fee waiver certificates to all Ethiopians with an NTD or from an NTD-affected family [43].

## Supporting information

S1 Table Caregiver demographics. (DOCX)

## References

[1] Deribe K, Tomczyk S, Tekola-Ayele F. Ten years of podoconiosis research in Ethiopia. PLoS Negl Trop Dis. 2013; 7(10): e2301 10.1371/journal.pntd.0002301 24130908 PMC3794913

[2] World Health Organisation (internet). Podoconiosis: endemic non-filarial elephantiasis. 2018. Acessed 14/02/2019. Available from: http://www.who.int/lymphatic_filariasis/epidemiology/podoconiosis/en/.

[3] Deribe K, Kebede B, Mengistu B, Negussie H, Sileshi M, Tamiru M, et al Podoconiosis in Ethiopia: From Neglect to Priority Public Health Problem. Ethiop Med J. 2017; 55(Suppl 1): 65–74. 28878431 PMC5582632

[4] Federal Democratic Republic of Ethiopia Ministry of Health. Second Edition of National Neglected Tropical Diseases Master Plan. Addis Ababa. 2016.

[5] Davey G, Newport M. Podoconiosis: the most neglected tropical disease? Lancet. 2007; 369(9565): 888–9. 10.1016/S0140-6736(07)60425-5 17368134

[6] Tekola Ayele F, Adeyemo A, Finan C, Hailu E, Sinnott P, Burlinson ND, et al HLA class II locus and susceptibility to podoconiosis. N Engl J Med. 2012; 366(13): 1200–8. 10.1056/NEJMoa1108448 22455414 PMC3350841

[7] Brooks J, Ersser SJ, Cowdell F, Gardiner E, Mengistu A, Matts PJ. A randomized controlled trial to evaluate the effect of a new skincare regimen on skin barrier function in those with podoconiosis in Ethiopia. Br J Dermatol. 2017; 177(5): 1422–31. 10.1111/bjd.15543 28374907

[8] Negussie H, Molla M, Ngari M, Berkley JA, Kivaya E, Njuguna P, et al Lymphoedema management to prevent acute dermatolymphangioadenitis in podoconiosis in northern Ethiopia (GoLBeT): a pragmatic randomised controlled trial. Lancet Glob Health. 2018; 6(7): e795–e803. 10.1016/S2214-109X(18)30124-4 29773516 PMC6562300

[9] National Podoconiosis Action Network (NaPAN) (internet). About Podoconiosis. 2013. Acccessed 14/02/2019. Available from: http://www.napanethiopia.org/index.php/about-podoconiosis/about-podoconiosis.

[10] Destas K, Ashine M & Davey G (2002) Prevalence of podoconiosis (endemic non‐filarial elephantiasis) in Wolaitta, southern Ethiopia. Tropical Doctor 32, 217–220. 10.1177/004947550303300410 14620426

[11] Tekola F, Mariam DH, Davey G. Economic costs of endemic non-filarial elephantiasis in Wolaita Zone, Ethiopia. Trop Med Int Health. 2006; 11(7): 1136–44. 10.1111/j.1365-3156.2006.01658.x 16827714

[12] Molla YB, Tomczyk S, Amberbir T, Tamiru A, Davey G. Patients' perceptions of podoconiosis causes, prevention and consequences in East and West Gojam, Northern Ethiopia. BMC Public Health. 2012; 12: 828 10.1186/1471-2458-12-828 23020758 PMC3519620

[13] Yakob B, Deribe K, Davey G. High levels of misconceptions and stigma in a community highly endemic for podoconiosis in southern Ethiopia. Trans R Soc Trop Med Hyg. 2008 5;102(5):439–44. 10.1016/j.trstmh.2008.01.023 18339411

[14] Deribe K, Cano J, Newport MJ, Pullan RL, Noor AM, Enquselassie F, et al The global atlas of podoconiosis. Lancet Glob Health. 2017; 5(5): e477–e9. 10.1016/S2214-109X(17)30140-7 28395836 PMC5390851

[15] Davey G, Bockarie M, Wanji S et al Launch of the International Podoconiosis Initiative. Lancet 2012;379 (9820):1004 10.1016/S0140-6736(12)60427-9 22423883

[16] Deribe K, Cano J, Giorgi E, Pigott DM, Golding N, Pullan RL, et al Estimating the number of cases of podoconiosis in Ethiopia using geostatistical methods. Wellcome Open Res. 2017; 2:78 10.12688/wellcomeopenres.12483.2 29152596 PMC5668927

[17] Addiss DG, Brady MA. Morbidity management in the Global Programme to Eliminate Lymphatic Filariasis: a review of the scientific literature. Filaria J. 2007; 6:2 10.1186/1475-2883-6-2 17302976 PMC1828725

[18] Eddy BA, Blackstock AJ, Williamson JM, Addiss DG, Streit TG, Beau de Rochars VM, et al A longitudinal analysis of the effect of mass drug administration on acute inflammatory episodes and disease progression in lymphedema patients in Leogane, Haiti. Am J Trop Med Hyg. 2014; 90(1): 80–8. 10.4269/ajtmh.13-0317 24218408 PMC3886433

[19] Prieto-Pérez L, Soriano Cea JJ, Górgolas Hernández-Mora M. Podoconiosis, a society and medical community neglected disease. Med Clin (Barc). 2015; 145(10): 446–51. 25726310 10.1016/j.medcli.2014.12.020

[20] Sikorski C, Ashine M, Zeleke Z, Davey G. Effectiveness of a simple lymphoedema treatment regimen in podoconiosis management in southern ethiopia: one year follow-up. PLoS Negl Trop Dis. 2010; 4(11): e902 10.1371/journal.pntd.0000902 21152059 PMC2994920

[21] Bekele K, Deribe K, Amberbir T, Tadele G, Davey G, Samuel A. Burden assessment of podoconiosis in Wayu Tuka woreda, east Wollega zone, western Ethiopia: a community-based cross-sectional study. BMJ Open. 2016; 6(9): e012308 10.1136/bmjopen-2016-012308 27670520 PMC5051403

[22] Getahun A, Ayele FT, Takele D, Ahrens C, Davey G. Burden of podoconiosis in poor rural communities in Gulliso woreda, west Ethiopia. PLoS Neglected Tropical Diseases. 2011; 5(6). 10.1371/journal.pntd.0001184 PMC3110157 21666795

[23] Thrush A, Hyder AA. The neglected burden of caregiving in low- and middle-income countries. Disabil Health J. 2014; 7(3): 262–72. 10.1016/j.dhjo.2014.01.003 24947567

[24] World Health Organization. World Report on Aging and Health. Geneva. 2015.

[25] Adelman RD, Tmanova LL, Delgado D, Dion S, Lachs MS. Caregiver burden: a clinical review. JAMA. 2014; 311(10): 1052–60. 10.1001/jama.2014.304 24618967

[26] Hotez PJ, Alvarado M, Basáñez MG, Bolliger I, Bourne R, Boussinesq M, et al The global burden of disease study 2010: interpretation and implications for the neglected tropical diseases. PLoS Negl Trop Dis. 2014; 8(7): e2865 10.1371/journal.pntd.0002865 25058013 PMC4109880

[27] Herricks JR, Hotez PJ, Wanga V, Coffeng LE, Haagsma JA, Basáñez MG, et al The global burden of disease study 2013: What does it mean for the NTDs? PLoS Negl Trop Dis. 2017; 11(8): e0005424 10.1371/journal.pntd.0005424 28771480 PMC5542388

[28] Litt E, Baker MC, Molyneux D. Neglected tropical diseases and mental health: a perspective on comorbidity. Trends Parasitol. 2012; 28(5): 195–201. 10.1016/j.pt.2012.03.001 22475459

[29] Family Health International. Qualitative Research Methods: A Data Collector’s Field Guide. USA; 2005.

[30] Given L (Ed). The SAGE encyclopedia of Qualitative Research Methods. California: Sage Publications; 2008.

[31] Braun V, Clarke V. Successful qualitative research: a practical guide for beginners. London: Sage; 2013.

[32] Bryman A. Social Research Methods. 5th Edition ed. Oxford: Oxford University Press; 2016.

[33] Hosseinpoor AR, Bergen N, Chatterji S. Socio-demographic determinants of caregiving in older adults of low- and middle-income countries. Age Ageing. 2013;42(3):330–8. 4 10.1093/ageing/afs196 23612865

[34] Hsieh HF, Shannon SE. Three approaches to qualitative content analysis. Qual Health Res. 2005; 15(9): 1277–88. 10.1177/1049732305276687 16204405

[35] Perera M, Whitehead M, Molyneux D, Weerasooriya M, Gunatilleke G. Neglected Patients with a Neglected Disease? A Qualitative Study of Lymphatic Filariasis. PLoS Negl Trop Dis. 2007; 1(2) 10.1371/journal.pntd.0000128 PMC2100378 18060080

[36] Tembei AM, Kengne-Ouaffo JA, Ngoh EA, John B, Nji TM, Deribe K, et al A Comparative analysis of Economic Cost of Podoconiosis and Leprosy on Affected Households in the Northwest Region of Cameroon. Am J Trop Med Hyg. 2018; 98(4): 1075–81. 10.4269/ajtmh.17-0931 29460727 PMC5899508

[37] Ton TG, Mackenzie C, Molyneux DH. The burden of mental health in lymphatic filariasis. Infect Dis Poverty. 2015; 4: 34 10.1186/s40249-015-0068-7 26229599 PMC4520254

[38] Bailey F, Mondragon-Shem K, Haines LR, Olabi A, Alorfi A, Ruiz-Postigo JA, et al Cutaneous leishmaniasis and co-morbid major depressive disorder: A systematic review with burden estimates. PLoS Negl Trop Dis. 2019; 13(2) 10.1371/journal.pntd.0007092 PMC6405174 30802261

[39] Chang HY, Chiou CJ, Chen NS. Impact of mental health and caregiver burden on family caregivers' physical health. Arch Gerontol Geriatr. 2010; 50(3): 267–71. 10.1016/j.archger.2009.04.006 19443058 PMC7114152

[40] Zergaw A, Hailemariam D, Alem A, Kebede D. A longitudinal comparative analysis of economic and family caregiver burden due to bipolar disorder. Afr J Psychiatry (Johannesbg). 2008; 11(3): 191–8. 19588042 10.4314/ajpsy.v11i3.30268

[41] Pai S, Kapur RL. The burden on the family of a psychiatric patient: development of an interview schedule. Br J Psychiatry. 1981; 138: 332–5. 7272637 10.1192/bjp.138.4.332

[42] Thara R, Padmavati R, Kumar S, Srinivasan L. Instrument to assess burden on caregivers of chronic mentally ill. Indian J Psychiatry. 1998; 40(1): 21–9. 21494438 PMC2964812

[43] Tamiru A, Tsegay G, Wubie M, Gedefaw M, Tomczyk S, Tekola-Ayele F. Podoconiosis patients' willingness to pay for treatment services in Northwest Ethiopia: potential for cost recovery. BMC Public Health. 2014; 14: 259 10.1186/1471-2458-14-259 24642085 PMC4234032

