# Supplementary material for: The impact of acute adenolymphangitis in podoconiosis on caregivers: A case study in Wayu Tuka woreda, Oromia, Western Ethiopia. ‘If she was healthy, I would be free.’
Source: PLoS Negl Trop Dis. 2019 Jul 8;13(7):e0007487. doi: 10.1371/journal.pntd.0007487 (PMC6638979; doi:10.1371/journal.pntd.0007487)
Supplement: S1 Table — (DOCX) [file pntd.0007487.s001.docx]

**S1 Table - Caregiver demographics**

| Participant number | Age | Gender | Occupational status | Marital status | Size of household | Occupation | Highest educational attain | Age of onset of podoconiosis (of patient) | Primary caregiver | Disease stage of podoconiosis (of patient) | Number of acute attacks per month (experienced by patient) |
| --- | --- | --- | --- | --- | --- | --- | --- | --- | --- | --- | --- |
| Participant 02cg | 35 | Male | Works full time | Married | 6 | Farmer and daily labourer | Incomplete primary education | 16-20 | Husband | 3 | 10 |
| Participant 03cg | 26 | Male | Works full time | Married | 5 | Farmer | Completed lower secondary education grades 9-10 | 5-10 | Husband | 3 | 4 |
| Participant 04cg | 17 | Male | Works full time | Never married | 7 | Student | Completed primary education grades 1-8 | Unknown | Son | 2 | 4 |
| Participant 05cg | 22 | Male | Works full time | Married | 5 | Daily labourer | Completed primary education grades 1-8 | 25-30 | Son-in-law | 2 | 7 |
| Participant 06cg | 16 | Female | Works full time | Never married | 6 | Student | Completed primary education grades 1-8 | Unknown | Granddaughter | 4 | 4 |
| Participant 07cg | 23 | Female | Works full time | Never married | 4 | Merchant | Unassigned | 25-30 | Daughter | 4 | 12 |
| Participant 08cg | 25 | Female | Works full time | Married | 5 | Farmer | No formal education | 30+ | Daughter | 2 | 3 |
| Participant 09cg | 40 | Female | Unable to work due to ill-health | Married | 7 | Farmer | Completed primary education grades 1-8 | 21-25 | Wife | 4 | 6 |
| Participant 10cg | 55 | Male | Works part-time | Married | 5 | Daily labourer | No formal education | 16-20 | Husband | 4 | 10 |
| Participant 11cg | 45 | Female | Works full time | Married | 7 | Farmer and merchant | Incomplete primary education | 16-20 | Sister-in-law | 3 | 5 |
| Participant 12cg | 38 | Male | Works full time | Married | 6 | Farmer | Incomplete primary education | 11-15 | Brother | 4 | 6 |
| Participant 13cg | 55 | Male | Works part-time | Married | 5 | Farmer | No formal education | 16-20 | Husband | 3 | 5 |
| Participant 14cg | 43 | Male | Works full time | Married | 5 | Farmer | Completed primary education grades 1-8 | Unknown | Husband | 3 | 5 |
